# Supplementary material for: Learning optimal integration of spatial and temporal information in noisy chemotaxis
Source: PNAS Nexus. 2024 Jun 14;3(7):pgae235. doi: 10.1093/pnasnexus/pgae235 (PMC11216223; doi:10.1093/pnasnexus/pgae235)
Supplement: pgae235_Supplementary_Data [file pgae235_supplementary_data.pdf]

PNAS nexus

Supporting Information for

Learning optimal integration of spatial and temporal information in noisy chemotaxis

Albert Alonso and Julius B. Kirkegaard

Corresponding: Julius B. Kirkegaard.
E-mail: juki@di.ku.dk

This PDF file includes:

Supporting text
Figs. S1 to S9
Tables S1 to S3
SI References

Supporting Information Text

1. Comparison with interpretable models

In this section, we evaluate the chemotactic efficiency of the trained policies by comparing them to simpler strategies that are straightforward in explaining the integration of memory and spatial gradient sensing.

We begin by defining a naive spatial policy $\tilde{\pi}$ where the steering of the swimming orientation is directly dictated by the strength of the receptor measurements. The naive optimal reorientation is given by

$$\phi = \mathrm{atan2}\left(\frac{\sin(\boldsymbol{\alpha}) \cdot \boldsymbol{\omega}}{\cos(\boldsymbol{\alpha}) \cdot \boldsymbol{\omega}}\right), \qquad [1]$$

where $\boldsymbol{\omega}$ are the contributions of each receptor to the decision and $\boldsymbol{\alpha}$ are the angles of the receptor position on the cell's surface with respect to the swimming direction (FIG.1A). This naive strategy is very susceptible to fluctuations in measurements and can sometimes be improved by restricting the reorientations to a certain ε. Thus, we consider policies of the form

$$a_t = \tilde{\pi}(s_t) = \frac{1}{\Delta t} \cdot \begin{cases} -\varepsilon & \text{if } \phi \leq -\varepsilon \\ \varepsilon & \text{if } \phi \geq \varepsilon \\ \phi & \text{otherwise.} \end{cases} \qquad [2]$$

Integrating measurements over time reduces the fluctuations in concentration measurements, as has also been shown experimentally [1]. Thus, we explore the possibility of cells relying on the average of previous measurements to set the change in orientation. The contribution of each sensor is then averaged by previous measurements as

$$\omega_t^{(i)} = \int_0^\infty \kappa(t')\, \hat{m}^{(i)}(t - t')\, \mathrm{d}t'. \qquad [3]$$

Here, $\hat{m}(t)$ are corrected measurements at time t. Directly using $m(t)$ completely ruins performance, as every time an action is performed, the information of previous measurements is no longer aligned with the cell orientation. To obtain optimal strategies, we use $\hat{m}(t)$, which is corrected by the action taken a_t, and thus only suffers from information decay due to rotational diffusion.

We begin by studying a uniform distribution, such as

$$\kappa(t) = \begin{cases} \frac{1}{T} & \text{for } t \leq T \\ 0 & \text{otherwise} \end{cases} \qquad [4]$$

where all previous measurements contribute the same up to T. Moreover, we consider the use of an exponentially decaying kernel

$$\kappa(t) = \frac{1}{T} e^{-t/T}, \qquad [5]$$

which gives more weight to newer measurements.

As seen in FIG. S1, the chemotactic efficiency of these models outperforms S and T when some rudimentary use of memory is allowed. We note that each reported value on the analytical strategies is evaluated with different ε, and only the best-performing one is shown. Nevertheless, we observe that a large memory timescale becomes counterproductive as the movement of the cell makes previous measurements irrelevant and only contributes noise to the decision. Despite the gain in efficiency, the optimal timescale for the proposed models is far from reaching the chemotactic efficiency of C.

We note that as $T \to 0$, S outperforms the explicit models. This can be explained by the freedom of S to dynamically control a non-linear equivalent of ε depending on the measurements. With this in mind, we investigate a new RL agent using the same neural network as S, but whose input is given by Eq. Eq. (3). Thus the integration of memory is fully controlled, but any non-linear action can be taken based on this input. We note that this again requires correcting previous inputs and special attention is given to the early parts of trajectories, such that the policy only averages over known measurements. FIG. S1 shows that this indeed outperforms S and T, but cannot reach the performance of C. This suggests that C is not just combining a *temporal average* with a *spatial strategy* but is also using elements of a *temporal strategy*.

Spermatozoa have recently been shown to exhibit a biphasic chemotactic strategy, in which there is a concentration-dependent switch between hyperactive phases, characterized by random changes in orientation, and more well-known chiral motion [2]. Presently, a switch between a temporal and a spatial strategy could achieve the best of the distinct time distributions of T and S in FIG.3B. We implement this by setting a cutoff particle count at which we switch from T to S. As a function of this threshold, an increase in chemotactic efficiency is observed, as shown in FIG. S1, but this also does not reach the efficiency achieved by C. Nevertheless, the increase in efficiency does suggest that the contribution of temporal and spatial may change dynamically with the concentrations.

Finally, we explore the possibility of designing an agent where the effective memory scale T is linearly dependent on the measurement concentration, as suggested in FIG.4B, such that

$$T = A\, \langle m_t \rangle + B \qquad [6]$$

We evaluate for different parameters of A, B, and ε on a uniform kernel. FIG S2 shows the chemotactic efficiency at different parameters A, with the best performant $B^*(A)$ and $\varepsilon^*(A)$.

The performance of this model is similar to that of a fixed uniform kernel. While the study of integrated gradients shows the amount of memory used, it does not reveal how this memory is used. In particular, here, we find that a simple uniform kernel is far from enough to reach optimal behavior.

References

1. RG Endres, NS Wingreen, Accuracy of direct gradient sensing by single cells. *Proc. Natl. Acad. Sci.* **105**, 15749–15754 (2008).
2. M Zaferani, A Abbaspourrad, Biphasic Chemokinesis of Mammalian Sperm. *Phys. Rev. Lett.* **130**, 248401 (2023).

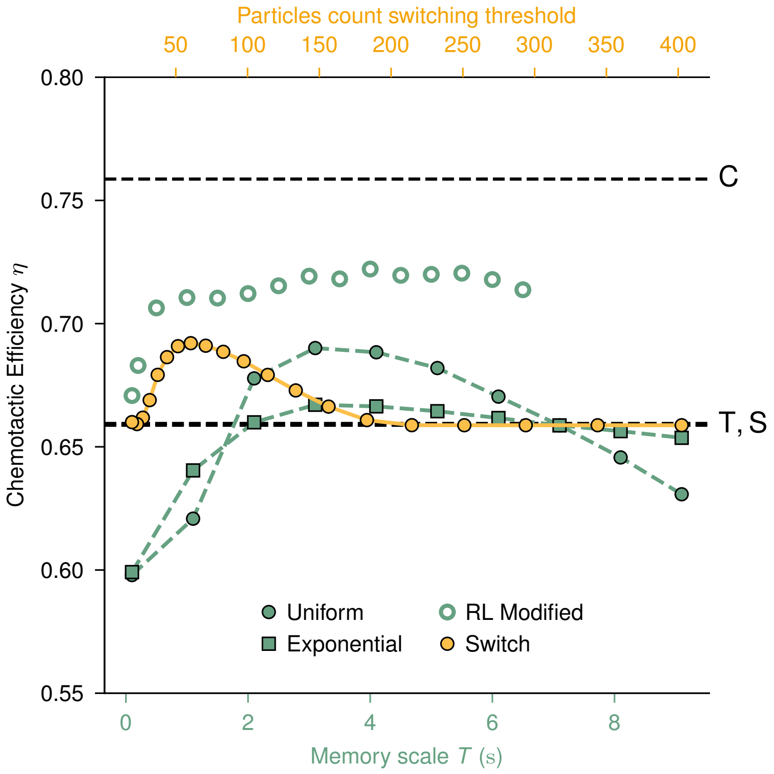

Fig. S1. Chemotactic efficiency of proposed explicit policies compared to the neural network policies found using reinforcement learning, at $R = 2\ \mu\mathrm{m}$. Green points are for policies that integrate measurements over time (lower axis), whereas orange points correspond to the policy achieved by switching between temporal and spatial strategies at a certain concentration threshold (upper axis).

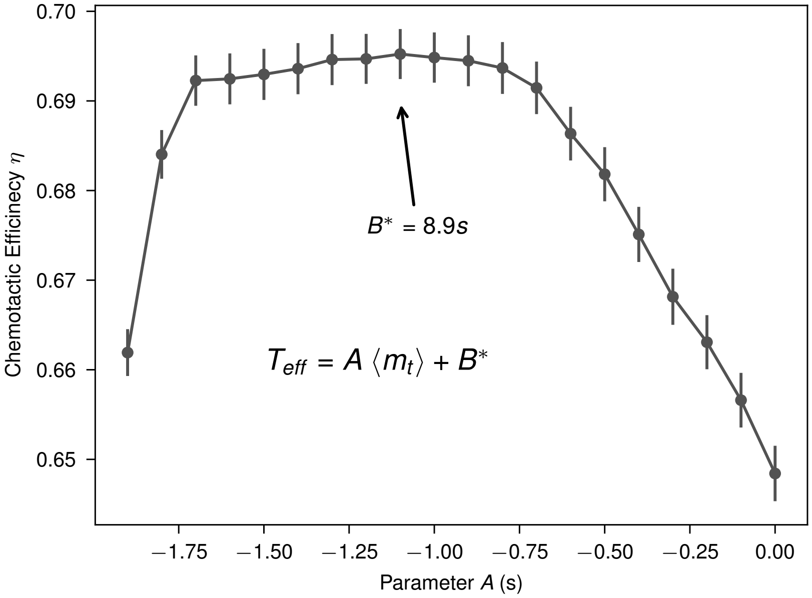

Fig. S2. Chemotactic efficiency of a policy that adjusts the memory time scale according to a linear dependency with the average strength of the measurements $T = A\langle m_t \rangle + B$. The efficiency is shown as a function of A, and B and ε are the optimal values for that A. The simulation parameters are the same as in FIG. S1.

Table S1. Default parameters used on the simulation of the environment during training and evaluation runs, if not stated otherwise.

Description	Symbol	Value	Unit
Number of receptors	K	5	–
Cell speed	v	5	$\mu\mathrm{m/s}$
Rotational diffusion coefficient	D_R	0.025	s^{-1}
Chemoattractant diffusion coefficient	D	100	$\mu\mathrm{m}^2/\mathrm{s}$
Chemoattractant decay rate	κ	0.1	$1/\mathrm{s}$
Time step	Δt	0.1	s
Lower limit concentration levels	C_q	16	$1/\mu\mathrm{m}^2$
Concentration levels	C_0	$\sim U(C_q, 10\,C_q)$	–
Threshold distance to the source (10th percentile of N)	δ	$-\log(0.9)/\sqrt{\kappa/D}$	$\mu\mathrm{m}$
Initial distance	d_0	$-\log(\sim U(0.3, 0.7))/\sqrt{\kappa/D}$	$\mu\mathrm{m}$
Initial orientation	θ_0	$\sim U(-\pi, \pi)$	rad

Table S2. Parameters used during training to train using our Proximal Policy Optimization implementation

Description	Symbol	Value
Number of parallel training environments	N_{envs}	4096
Total number of simulation steps	N_{steps}	10^{10}
Simulation duration steps	$t_{\mathrm{max}}/\Delta t$	256
Maximum gradient norm clipping		0.5
Learning rate	η	$3 \cdot 10^{-4}$
Number of epochs	N_{epoch}	8
Number of mini batches	N_{batches}	8
Epsilon clipping	ε	0.2
Entropy coefficient	$\hat{\sigma}_s$	0.01
Critic coefficient	$\hat{\sigma}_c$	0.5
Advantage Discount	γ	1.0
Generalized advantage coefficient	λ	1.0
Number of hidden layers	K	2
Hidden layers size	L	64
Number of hidden state cells	M	25
Minimum variance in output	σ_{min}	0.05
Maximum variance in output	σ_{max}	1.0

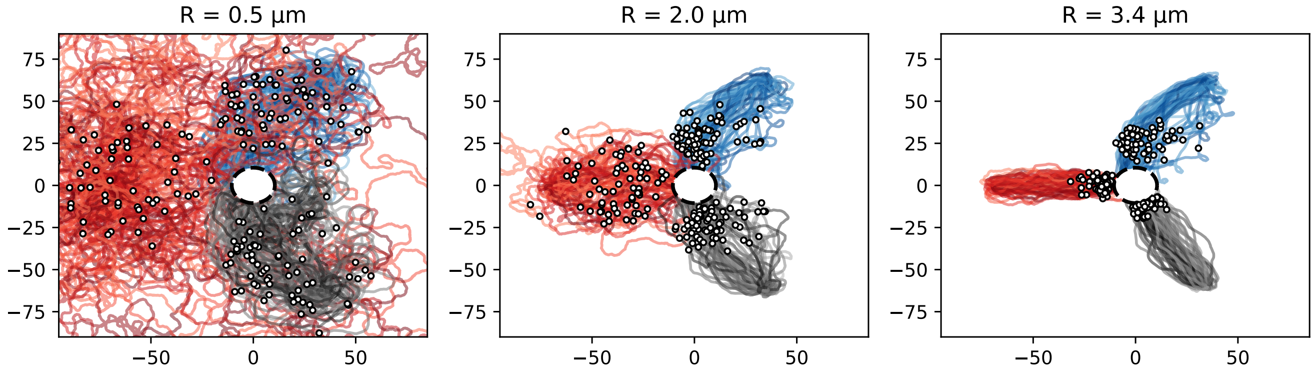

Fig. S3. Visual trajectories of the trained agents at different sizes. The three variants are displayed: Combined (black), Spatial (red), and Temporal (blue). The dot represents the cell position at $t = 12.5$ s.

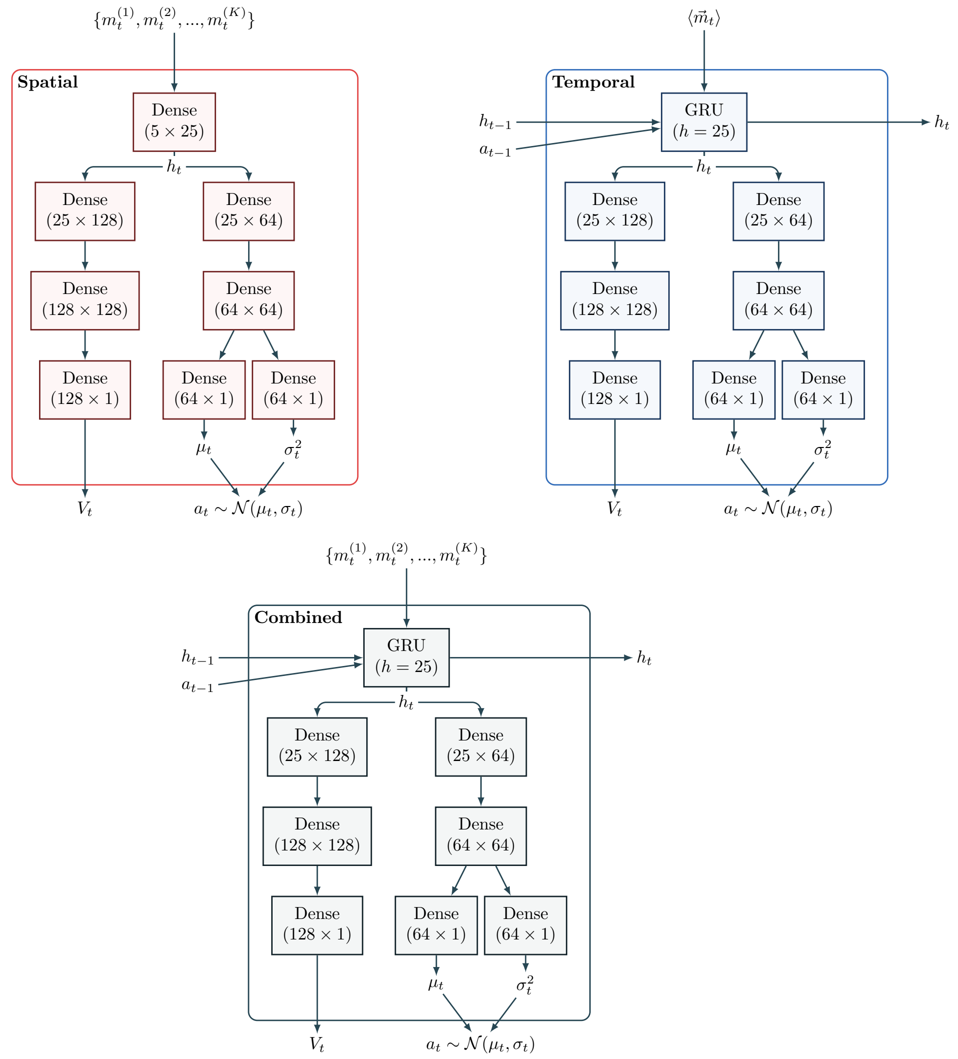

Fig. S4. Detail description of the three Neural Networks Architectures used. $\tanh$ is used as an activation function between layers, and σ is clipped to constrain its domain to be $\sigma \in [0.05, 1.0]$. The input to the reccurent layers (h_{t-1} and a_{t-1}) are concatenated.

Table S3. Summary of the parameter count on each of the variants for the action branch.

Variant	Number of Parameters	Comment
Combined	8379	
Temporal	8379	Average input is duplicated K times
Spatial	6102	GRU is replaced by Matrix

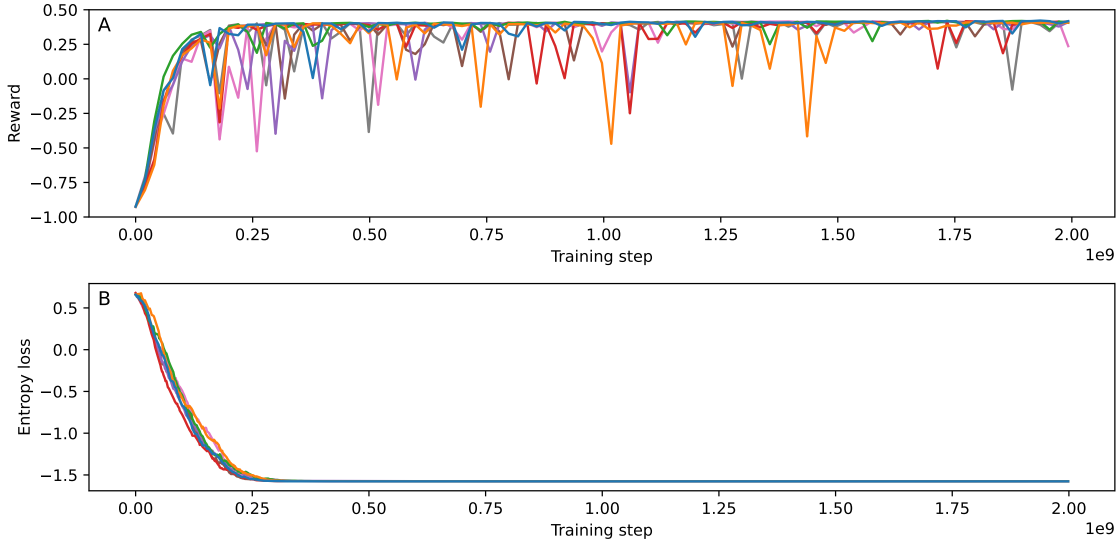

Fig. S5. A. Eight independent training of a policy at $R = 2.0\ \mu$m showing convergence to the same reward levels. Sudden drops in reward are a common problem in reinforcement learning, but we observe a quick return to the reward ceiling after each jump. **B**. Entropy loss, defined as the negative entropy $H_{\text{loss}} = -H = -\left(\frac{1}{2} + \frac{1}{2}\log(2\pi\sigma^2)\right)$, shows convergence towards a deterministic policy in PPO (to maintain exploration, σ is not permitted to reach exactly zero).

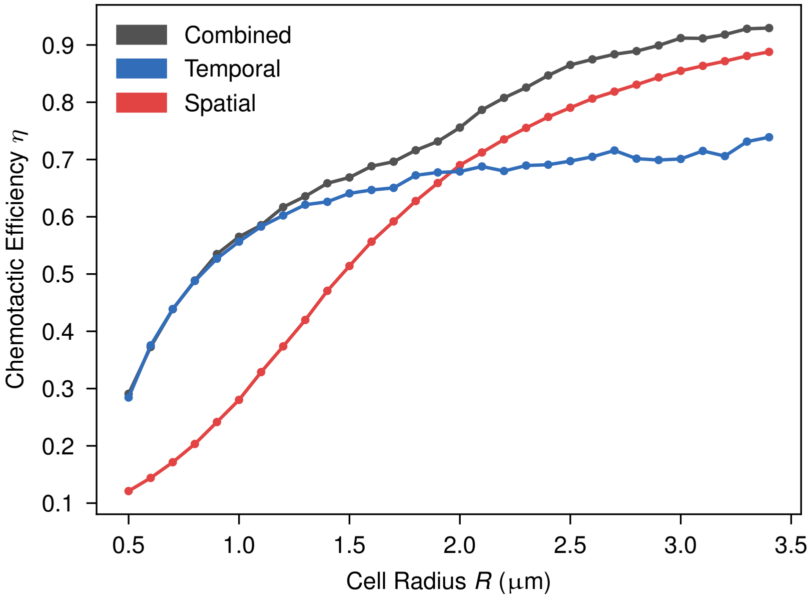

Fig. S6. Chemotactic efficiency as a function of cell size. We model the rotational diffusion of the agent as $D_{rot} = \left(\frac{a}{R}\right)^3$, with $a = 0.315\ \mu m\ s^{-4}$. Similar results (transition) are observed, with the main difference being at smaller sizes where the D_{rot} worsens the efficiency of all strategies.

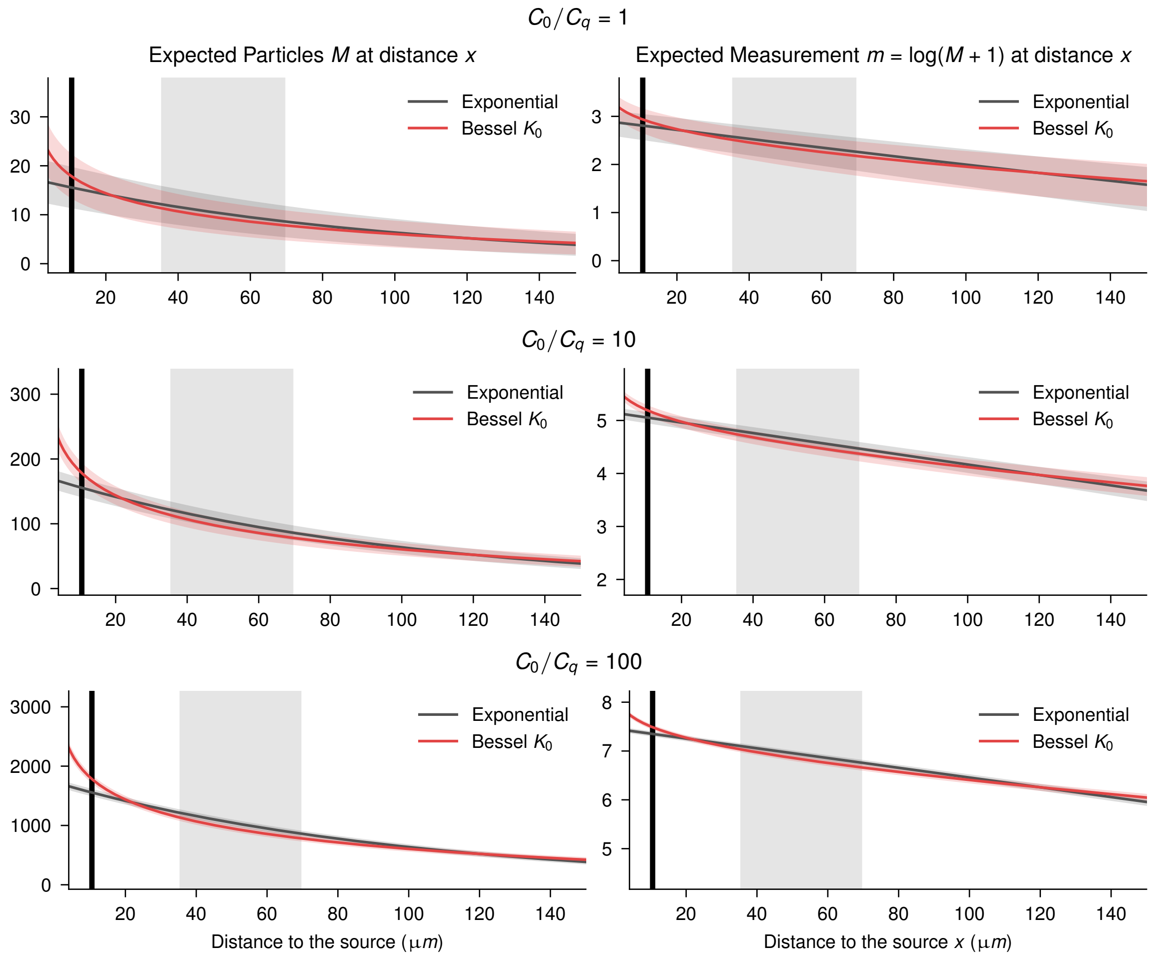

Fig. S7. Concentration profiles at different amounts of concentration levels C_0/C_q. The standard deviation of the expected values at different distances from the source is also shown to indicate the noise in the measurements. The thick black line represents δ, the closest the cells will be from the source, and the gray region indicates the initial distance region where the cell may start an episode. Both the Modified Bessel of second kind function and the exponential are plotted to showcase their similarity in the region of interest.

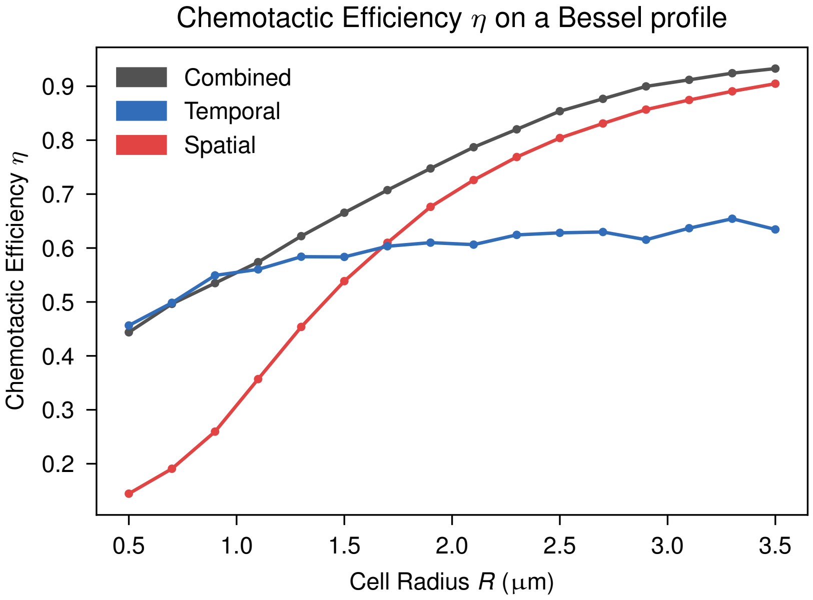

Fig. S8. Chemotactic efficiency while using a concentration profile $c(x) \propto K_0(\lambda x)$.

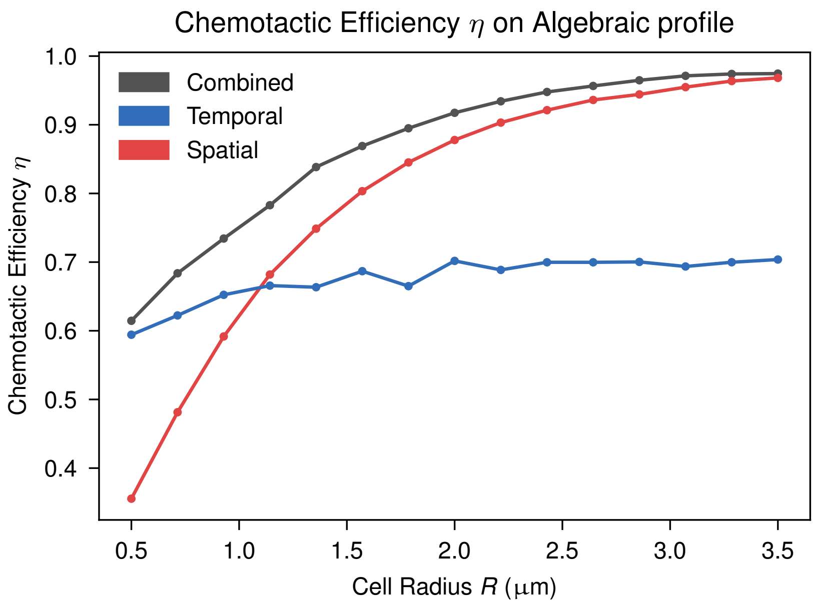

Fig. S9. Chemotactic efficiency with and algebraic concentration profile, i.e. $c(x) \propto 1/x$.
